# Impact of preconceptional serum thyroid stimulating hormone values ranging between 2.5 and 4.5 mIU/L on live birth rates following ovulation induction and intrauterine insemination treatment for unexplained infertility

**DOI:** 10.1186/s12905-021-01299-0

**Published:** 2021-04-19

**Authors:** Lale Susan Karakis, Huseyin Kiyak, Berfin Okmen, Cagdas Ozdemir, Engin Turkgeldi

**Affiliations:** 1 Bahceci IVF Centre, Hakkı Yeten Cad. 11/M3 Terrace Fulya, 34365 Sisli, Istanbul Turkey; 2 Istanbul Health Sciences University, Kanuni Sultan Suleyman Training and Research Hospital, Istanbul, Turkey; 3 Akademi Hospital, Gaziantep, Turkey; 4 Tunceli State Hospital, Tunceli, Turkey; 5 Koc University Hospital, Istanbul, Turkey

**Keywords:** Unexplained infertility, Intrauterine insemination, Subclinical hypothyroidism, Ovulation induction, Ovarian stimulation, TSH

## Abstract

**Background:**

Contrary to overt hypothyroidism, the true impact of subclinical hypothyroidism on fertility has not been well established. This study aimed to investigate whether serum thyroid stimulating hormone (TSH) values between 2.5 and 4.5 mIU/L are associated with lower pregnancy rates compared to TSH levels between 0.3 and 2.5 mIU/L in women undergoing ovulation induction with gonadotropins and intrauterine insemination (IUI) for unexplained infertility.

**Methods:**

Medical records of couples with unexplained infertility who underwent IUI treatment between January 2013 and December 2018 were reviewed retrospectively. Cycle characteristics and pregnancy outcomes of patients with serum TSH levels between 0.3–2.5 mIU/L and 2.5–4.5 mIU/L were compared. Primary outcome measures were clinical pregnancy and live birth rate. Secondary outcome measures were total dose of gonadotropin administration, duration of ovulation induction and miscarriage rate.

**Results:**

A total of 726 euthyroid women who underwent 1465 cycles of ovulation induction with gonadotropins and IUI were included in the analyses. Patient and cycle characteristics of the two study groups were similar. No statistically significant differences could be detected in the clinical pregnancy (*p* = 0.74) and live birth rates (*p* = 0.38) between the two groups. Duration of ovulation induction, total gonadotropin dosage, number of follicles > 17 mm on the trigger day and the miscarriage rates were similar in the two groups.

**Conclusion:**

In euthyroid women undergoing ovulation induction with gonadotropins and IUI for unexplained infertility, the range of preconceptional serum TSH values between 2.5 and 4.5 mIU/L is not associated with lower pregnancy rates when compared to TSH levels between 0.3 and 2.5 mIU/L.

## Background

Worldwide prevalence of infertility is estimated to range between 8 and 12% in reproductive aged couples [1]. In up to 30% of infertile couples, no underlying aetiology can be identified [2]. Ovulation induction and intrauterine insemination (IUI) is usually the first treatment modality offered to patients with unexplained infertility before more invasive and expensive treatments as in vitro fertilization are considered.

Thyroid disorders are the most common endocrine diseases encountered in women of reproductive age [3]. Overt hypothyroidism, defined as elevated TSH with low free T4 (fT4) levels, is known to be associated with ovulatory problems, infertility and adverse pregnancy outcomes including preeclampsia, preterm delivery, placental abruption and foetal neural developmental defects [4]. Therefore, the concept that overt hypothyroidism during pregnancy or in the pre-conceptional period necessitates treatment has been widely accepted. Similarly, subclinical hypothyroidism (SH), defined as elevated TSH in the presence of normal serum free thyroxine levels, has been found to be associated with adverse pregnancy outcomes [5]. SH has also been found to be more prevalent in infertile patients, especially in those with unexplained infertility [6]. However, the true impact of SH on fertility has not been well established since varying upper TSH threshold values have been used for defining subclinical hypothyroidism in related studies over the course of years.

The normal range of thyroid hormones are known to vary with age, geographical location, and ethnicity of the patient population [7]. If available, it is recommended to use local thyroid function test thresholds defined by a given laboratory [4]. For non-pregnant women, the upper TSH limits range between 4 and 5 mIU/L in most laboratories [8]. For pregnant women, due to changes in thyroid hormone physiology after conception, the use of thyroid function test thresholds defined specifically for each trimester of pregnancy have been recommended [9]. For patients attempting pregnancy or undergoing infertility treatment, it has been advised to use TSH levels specific to the first trimester of pregnancy in an effort to decrease the time to achieve conception and prevent development of overt hypothyroidism during pregnancy [4].

Whether TSH levels between 2.5 and 4.5 mIU/L have a negative impact on fertility or pregnancy outcomes is still a matter of debate, as data on the optimal normal upper limit of TSH above which treatment should be offered is controversial.

The aim of this study was to determine if there is a difference in the treatment outcome of euthyroid patients with TSH levels below 2.5 mIU/L and those between 2.5 and 4.5 mIU/L undergoing ovulation induction (OI) and IUI treatment for unexplained infertility.

## Methods

### Patients

This retrospective study was carried out at the Kanuni Sultan Suleyman Research and Training Hospital affiliated with the Health Sciences University in Istanbul, Turkey. Ethics approval was obtained from the local Institutional Review Board of the hospital. Medical records of couples with unexplained infertility who underwent ovulation induction and intrauterine insemination at the infertility clinic of the hospital from January 2013 to December 2018 were reviewed.

Women with unexplained infertility between the ages of 20 and 40 whose TSH and fT4 levels were within the normal range (0.27–4.5 mIU/ml) were included into the study. Patients on thyroid medication or with a history of thyroid disease, known thyroid autoimmunity (positive for anti-TPO or anti TG antibodies), body mass index (BMI) above 35 kg/m^2^ (Class 2 obesity) or below 18 kg/m^2^, antral follicle count (AFC) of less than 8 or more than 24, polycystic ovary syndrome, male factor infertility, endometriosis, tubal pathology or other endocrine or chronic systemic diseases were excluded from the study. Patients with cancelled cycles or timed intercourse cycles were also excluded from the study.

All female patients underwent routine fertility evaluation including day 3 or 4 serum hormone measurements (FSH, E2, TSH, fT4 and prolactin), mid-luteal progesterone measurements to confirm ovulatory cycles, transvaginal ultrasonography for antral follicle count and uterine imaging and a hysterosalpingogram to evaluate tubal patency. Semen samples were obtained from male partners and evaluated according to the WHO 2010 criteria [10].

Couples with normal ovarian reserve tests, sperm parameters, mid-luteal progesterone levels confirming ovulatory cycles and hysterosalpingographic evidence of bilateral tubal patency and normal uterine anatomy who could not conceive despite regular sexual intercourse for at least one year, or for six months in women 35 and older, were classified to have unexplained infertility. Male partners who had a total motile sperm count of at least 10 million were included to the study. All women had a progesterone level of > 3 ng/mL on the 21th day of the ovulatory cycle. The patients were tested for serum TSH and fT4 levels as part of the routine infertility checkup. The maximum length of time between thyroid hormone testing and start of treatment would be 1 month.

Patients were divided into two groups according to their TSH levels. Those with TSH levels under 2.5 mIU/L were assigned to group 1 and those with TSH levels above 2.5 mIU/L and ≤ 4.5 mIU/L were assigned to group 2.

### Thyroid assays

Normal range of TSH was calculated by the laboratory of the hospital as 0.27–4.5 mIU/ ml. All analyses of serum parameters were conducted at the ISO-certified central laboratory of the Kanuni Sultan Suleyman Training and Research Hospital, Health Care University of Health Sciences, Istanbul, Turkey, using commercially available Elecsys electrochemiluminescence immunoassays on a Cobas 6000 immunoanalyzer (Roche Diagnostics, Mannheim, Germany). The inter and intra assay coefficient of variations were < 2% and < 6.5% for TSH and the TSH measuring range was 0.005–100 μIU/mL.

### Ovulation induction and intrauterine insemination

Patients underwent transvaginal ultrasonography on the second or third day of their menstrual cycle. If no cysts were present, stimulation was initiated using subcutaneous recombinant follicle stimulating hormone (rFSH) injections (Gonal-F; Serono, Istanbul, Turkey) with a starting dose of 50–75 IU/day based on patient characteristics such as age, AFC and BMI. Ovarian response was monitored with serial transvaginal ultrasonography and medication doses and duration of stimulation were adjusted accordingly. Ovulation was triggered with 5,000–10,000 IU of human chorionic gonadotropin (hCG) (Choriomon; IBSA, Turkey) when one or two follicles reached a mean size of 18 mm. If more than three follicles reached a size of 18 mm or more, the cycle was cancelled to prevent multiple pregnancies.

Semen samples were obtained 2 h before the IUI procedure following 2–3 days of sexual abstinence. The samples were processed using the swim-up technique and stored at room temperature until the insemination procedure. IUI was performed 36 h after the hCG injection, using a soft tip catheter. Patients were asked to rest in the supine position for 15 min following IUI. Vaginal progesterone gel (Crinone 8%; Merck Serono, Turkey) was administered twice daily for luteal support from the day after IUI until the 9th week of pregnancy.

Serum beta chronic gonadotrophin (ß-hCG) levels were measured 12 days after insemination. Levels above 10 mIU/ml were considered positive and measurements were repeated 48 h after a positive pregnancy test to determine the ß-hCG doubling time.

### Outcome measures

The study groups were compared in terms of patient characteristics, hormone profiles, semen analyses and cycle characteristics. The primary outcomes of the study were clinical pregnancy and live birth rates. Clinical pregnancy was defined as the ultrasound visualization of foetal cardiac activity at 7 weeks of gestation. Live birth was defined as the delivery of a viable baby past the 24th week of pregnancy.

Secondary outcome measures were total dose of gonadotropin administration, duration of ovulation induction and miscarriage rate.

### Statistical analysis

SPSS version 18 (SPSS Inc.) was used for statistical analysis. The normality of data was assessed by Shapiro–Wilk test. Continuous variables were presented as median (minimum–maximum) with respect to data distribution. Categorical variables were presented as frequency (percentage). Continuous data were compared using the Mann–Whitney-U test. The Chi-Square or Fisher exact tests were used for the comparison of categorical variables. *P* values lower than 0.05 were considered statistically significant.

## Results

A total of 726 euthyroid women with unexplained infertility who underwent 1465 cycles of ovulation induction and IUI were included into the analyses. The median age of the subjects was 29 (19–40) years and the median TSH level was 1.7 (0.27–4.5) mIU/L. Of the 726 women involved in the study, 543 (74%) had TSH levels between 0.27–2.5 mIU/L (group 1) and 183 (26%) women had TSH levels between 2.51–4.5mIU/L (group 2). No statistically significant difference was found with respect to age, BMI, duration of infertility, antral follicle count, total motile sperm count, type of infertility (primary or secondary), history of pelvic surgery or smoking habits between the two groups. Comparison of patient characteristics of the two groups is presented in Table 1.

**Table 1:** Comparison of patient characteristics among the two study groups

	TSH 0.27–2.5 mIU/L n = 543 1110 cycles	TSH 2.51–4.5 mIU/L n = 183 355 cycles	
Age (years)*	29 (19–40)	29 (19–40)	0.07
Age range			
< 35	476 (87.6%)	160 (87.4%)	0.85
≥ 35	67 (12.4%)	23 (12.6%)	
Body mass index (kg/m^2^)*	24.2 (18.2–34.8)	23.4 (18.1–34.8)	0.08
Body mass index range			
< 25	316 (58.2%)	119 (65.0%)	0.67
25–29.9	182 (33.5%)	53 (28.9%)	
≥ 30	45 (8.2%)	11 (6.1%)	
Primary infertility	466 (85.8%)	303 (85.5)	0.92
Infertility duration (years)*	4.02 (2–9)	4.44 (2–10)	0.38
Infertility duration range			
< 3 years	165 (30.5%)	57 (31.1%)	0.78
3–5 years	212 (39.0%)	79 (43.1%)	
> 5 years	166 (30.5%)	47 (25.6%)	
History of pelvic surgery	77 (14.1%)	27 (14.7%)	0.84
Smokers, n	104 (19.3%)	36 (19.8%)	0.87
Smoking ranges			
1–5 cigarettes/day	42 (7.7%)	12 (6.5%)	0.71
6–10 cigarettes/day	45 (8.2%)	18 (9.8%)	
> 10 cigarettes/day	17 (3.1%)	6 (3.2%)	
Alcohol use, n	8 (1.4%)	4 (2.1%)	0.547
Basal assessment on day 2			
FSH level (IU/L)*	6.8 (2.4 -9.9)	6.8 (1.8–10.0)	0.801
Prolactin, ng/mL *	14.0 (4–30)	14.1 (5–30)	0.100
LH level (IU/L) *	4.8 (0.8–40.0)	5.1 (1.4–41.0)	0.091
Antral follicle count, n*	9 (8–23)	9 (8–24)	0.128
Total motile sperm count (millions) *	43 (10–346)	41 (10–330)	0.835

TSH, thyroid stimulating hormone; FSH, follicle stimulating hormone; LH, luteinizing hormone

*Values are median (25th–75th percentile)

The overall clinical pregnancy and live birth rates of the study population were 124 (7.8%) and 115 (8.4%) per cycle, respectively. No statistically significant difference was found between the clinical pregnancy rate of subjects in group 1 and those in group [96 (8.6%) vs 28 (7.9%), *p* = 0.74]. Likewise, live birth rates were similar between group 1 and group 2 [90 (8.1%) vs 25 (7.1%), *p* = 0.38]. The duration of ovulation induction, total gonadotropin dosage, number of follicles > 17 mm on the trigger day and the miscarriage rate were similar in the two study groups. Cycle characteristics and pregnancy outcomes of the two study groups are compared in Table 2.

**Table 2:** Comparison of treatment characteristics and pregnancy outcomes between the two study groups

	TSH 0.27–2.5 mIU/L n = 543 1110 cycles	TSH 2.51–4.5 n = 183 355 cycles	
Duration of ovulation induction (days)*	9 (5–12)	9 (5–11)	0.34
Total gonadotrophin dose used (IU)*	525 (262.5–5625)	546 (130–6950)	0.37
Number of follicles > 17 mm on triggerday, n*	1 (0–6)	1 (0–6)	0.65
Clinical pregnancy, n (%cycle)	96 (8.6%)	28 (7.9%)	0.74
Live birth, n (%cycle)	90 (8.1%)	25 (7.1%)	0.38
Miscarriage rate(cycle %)	6 (6.25%)	3 (10.7%)	0.46

TSH, thyroid stimulating hormone; FSH: follicle stimulating hormone; LH: luteinizing hormone

*Values are median (25th–75th percentile)

## Discussion

In the present study, no difference was detected in pregnancy outcomes between euthyroid women with TSH levels below 2.5 mIU/ml and women with TSH levels between 2.5 and 4.5 mIU/ml following ovulation induction and IUI for unexplained infertility.

The recommended upper TSH threshold levels for defining SH have changed considerably over the last decade. In 2012, the American Association of Clinical Endocrinologists and the American Thyroid Association (ATA) recommended lowering the TSH normal upper limit to 2.5 mIU/L in the first trimester, 3 mIU/L in the second trimester and 3.5 mIU/L in the third trimesters of pregnancy to define SH in the absence of population based reference ranges [9]. However, subsequent studies failed to show improved pregnancy outcomes with thyroid hormone replacement when a TSH cut-off value of 2.5 mIU/L was used for SH. In 2015, the Practice Committee of the American Society for Reproductive Medicine guidelines stated that scientific evidence was insufficient to associate adverse pregnancy outcomes with TSH levels between 2.5 and 4 mIU/L [8]. Instead, the beneficial effects of levothyroxine treatment on pregnancy outcomes were more evident when an upper TSH limit of 4 mIU/L was used for initiating treatment [11]. Consequently, 2017 ATA guidelines revised the upper reference limit for TSH, and advised 4.0 mU/L to be used as a cut-off level when defining SH [4]. The results of the present study support these findings since no difference in fertility rates could be detected between high normal and low normal TSH levels below 4.5mIU/L in euthyroid patients with unexplained infertility undergoing ovulation induction and IUI.

Recent studies restricting the upper threshold of TSH to a range of 4–4.99 have not found a significant difference in pregnancy rates of patients undergoing IUI with TSH levels either below or above 2.5 mIU/ml [12, 13]. In their study of 156 women, Turgay et al. compared the IUI outcomes of anti-TPO negative patients with unexplained infertility and found no difference among patients with TSH levels below 2.5 mIU/L and those with TSH levels between 2.5 and 4 mIU/L [14]. Similarly, Karmon et al. reported that preconception TSH levels in the high normal range (2.5–4.99 mIU/ml) had no significant effect on clinical pregnancy rates in infertile patients undergoing IUI treatment for various indications, including male factor infertility, diminished ovarian reserve, anovulation and unexplained infertility [15]. Repelaer van Driel-Delprat et al. studied the effects of TSH levels in a heterogenous group of euthyroid women who underwent up to a maximum of six cycles of IUI and found no difference in the rate of live birth, clinical and ongoing pregnancy, or pregnancy loss in patients with TSH levels between 2.35 and 4.5 mIU/L compared to women with TSH levels below 2.5 mIU/L [16].

Independent from thyroid hormone status, thyroid autoimmunity has been linked with an increased risk of pregnancy loss due to miscarriage or premature delivery [17]. Because anti-TPO positive euthyroid women are under increased risk for developing hypothyroidism over time, some authors believe it is prudent to start levothyroxine treatment when TSH levels are above 2.5 mIU/L during pregnancy [18]. However, recent reports comparing pregnancy outcomes in euthyroid infertile patients undergoing IUI or in vitro fertilization (IVF) have failed to demonstrate any significant effect of anti TPO positivity on treatment outcomes [19, 20]. Furthermore, levothyroxine treatment in anti-TPO positive euthyroid patients undergoing assisted reproductive technology (ART) treatments has not been found to decrease miscarriage rates or increase live birth rates [21]. There is currently no consensus on the management of euthyroid (TSH ≤ 4 mIU/L), anti-TPO positive women; however, if hormone treatment is not started, it is advised to monitor TSH levels closely during pregnancy for the development of hypothyroidism [4].

The ATA guidelines state there is insufficient evidence to recommend treatment for euthyroid women attempting natural pregnancy with TSH levels above 4 mIU/L, due to the lack of randomized controlled trials [4]. On the other hand, for infertile patients undergoing ART, two randomized controlled trials have demonstrated improved pregnancy outcomes with levothyroxine treatment in euthyroid women with TSH levels above 4 mIU/L [22, 23]. No such benefit of treatment was found for patients undergoing ART with TSH levels below 4.78 mIU/L [24]. Nevertheless, TSH concentrations have been shown to increase during controlled ovarian stimulation for IVF/ICSI (intracytoplasmic sperm injection) cycles in response to supra-physiologic estradiol levels [22]. Consequently, patients with high normal TSH levels undergoing ART treatment may be under greater risk for development of overt hypothyroidism when compared with patients attempting natural pregnancy. This may be the reason why, although most studies show no difference in ART outcomes among patients with TSH levels below 2.5 mIU/L and those with TSH levels between 2.5 and 5 mIU/L, the current ATA guidelines recommend treatment for TSH levels above 2.5 mIU/L in women undergoing IVF or ICSI treatment [4]. Data on the effect of ovulation induction with gonadotropins on TSH levels during IUI treatment is scarce.

It is important to keep in mind that although subclinical hypothyroidism can progress to overt hypothyroidism over the course of years, it is unlikely to do so during immediate pregnancy [25]. There is evidence that overtreatment with thyroid hormones is not only unnecessary, but also may increase the incidence of several complications. A retrospective study noted that levothyroxine treatment for TSH levels between 2.5 and 4 mIU/ml during pregnancy did not decrease the incidence of pregnancy loss but increased the risk of preterm delivery, diabetes and preeclampsia [11]. Korevaar et al. studied the association between free thyroxine levels measured before 18 weeks of pregnancy in women with normal serum TSH levels (0.05–4.09 mIU/ml) and the IQ scores of their offspring. They found that not only low, but also high levels of maternal free thyroxine concentrations were associated with low IQ scores in children at 6 years of age. They speculated that women treated for subclinical hypothyroidism during pregnancy might especially be prone to the detrimental effects of overtreatment with levothyroxine on the neurodevelopment of their offspring [26].

Considering the possible adverse effects of overtreatment and the lack of evidence on the detrimental effects of TSH levels between 2.5 and 4.5mIU/ml on fertility outcomes of euthyroid women undergoing ovulation induction and IUI, it seems unnecessary to offer levothyroxine treatment to such patients. There may be differences in thyroid hormone physiology in patients undergoing IVF/ICSI treatment and it may be prudent to study such patients as a separate group from those attempting natural pregnancy or undergoing IUI.

One of the strengths of this study is the large sample size and inclusion of a relatively homogenous patient population of women with unexplained infertility who underwent ovulation induction using only gonadotropins. The limitations of the study include its retrospective nature and the lack of information on the anti-TPO status of patients. Furthermore, the effect of ovulation induction with gonadotropins on TSH levels during IUI treatment could not be determined as serial TSH measurements were not made during the treatment. Also, the lack of information on the obstetric outcomes of patients with different TSH levels, which would have led further insight into the true impact of subclinical hypothyroidism on reproduction, is another limitation of the study.

## Conclusions

Findings of the present study indicate that TSH levels below 2.5 mIU/L and between 2.5 and 4.5 mIU/L do not have a critical impact on pregnancy outcomes in women undergoing IUI for unexplained infertility. Future well designed randomized controlled trials will help to determine the optimal range of pre-conceptional TSH levels for different populations of patients seeking pregnancy and those who will benefit most from levothyroxine treatment in terms of fertility treatment and pregnancy outcomes.

## Data Availability

The datasets used and/or analysed during the current study are available from the corresponding author on request.

## Abbreviations

TSH: Thyroid stimulating hormone
IUI: Intrauterine insemination
SH: Subclinical hypothyroidism
OI: Ovulation induction
Anti TPO: Anithyroidperoxidase antibody
Anti TG: Antithyroglobulin antibody
BMI: Body mass index
AFC: Antral follicle count
FSH: Follicle stimulating hormone
E2: Estradiol
fT4: Free thyroxine
WHO: World Health Organization
rFSH: Recombinant follicle stimulating hormone
hCG: Human chorionic gonadotropin
βhCG: Beta human chorionic gonadotropin
SPSS: Statistical package for the social sciences
ATA: American Thyroid Association
IVF: In vitro fertilization
ART: Assisted reproductive technology
ICSI: Intracytoplasmic sperm injection
IQ: Intelligence quotient
